# Computationally Efficient Locally Adaptive Demosaicing of Color Filter Array Images Using the Dual-Tree Complex Wavelet Packet Transform

**DOI:** 10.1371/journal.pone.0061846

**Published:** 2013-05-03

**Authors:** Jan Aelterman, Bart Goossens, Jonas De Vylder, Aleksandra Pižurica, Wilfried Philips

**Affiliations:** IPI-TELIN-IMINDS, Ghent University, Ghent, Belgium; University of Adelaide, Australia

## Abstract

Most digital cameras use an array of alternating color filters to capture the varied colors in a scene with a single sensor chip. Reconstruction of a full color image from such a color mosaic is what constitutes demosaicing. In this paper, a technique is proposed that performs this demosaicing in a way that incurs a very low computational cost. This is done through a (dual-tree complex) wavelet interpretation of the demosaicing problem. By using a novel locally adaptive approach for demosaicing (complex) wavelet coefficients, we show that many of the common demosaicing artifacts can be avoided in an efficient way. Results demonstrate that the proposed method is competitive with respect to the current state of the art, but incurs a lower computational cost. The wavelet approach also allows for computationally effective denoising or deblurring approaches.

## Introduction

**Figure 1:**
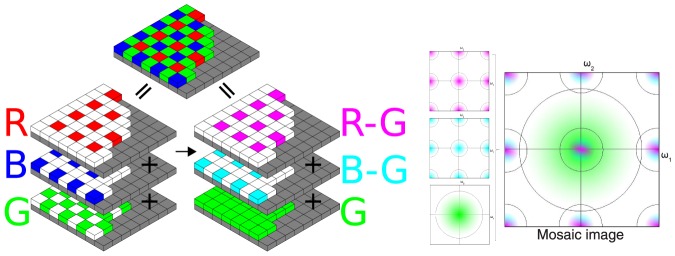
Interpretation of the mosaic image as the sum of three subsampled bands or as the sum of fully sampled green and two subsampled color difference bands (left) and the corresponding power spectral densities of the color difference interpretation (right). The mixed cyan and magenta signifies a superposition of red and blue color difference spectral energy.

There is a large scientific and industrial interest in color filter array (CFA) interpolation of Bayer arrays [1] (i.e. demosaicing). The Bayer array is a monochrome capture system of light sent through a periodic system of color filters as shown in Figure 1. Converting this data to a color image is called demosaicing, it is is often performed within the limited computational capabilities of digital cameras, so computationally and memory efficiency is an important requirement for a practical demosaicing algorithm. A first class of techniques are those that use interpolation, the most simple examples are nearest neighbor or bilinear interpolation, but these techniques suffer from significant artifacts due to frequency crosstalk. More advanced techniques greatly reduce these artifacts, at the cost of a higher computational complexity. The method of Zhang and Wu [2] does this by fusing directional minimum mean squared error (MMSE) estimates according to an edge adaptive criterion. In [3], [4], alternating projections are used to enforce natural image prior models, such as inter-channel spectral correlations. Paliy et al. [5], [6] present a method which carefully uses a combination of local polynomial approximation (LPA) interpolation with intersections of confidence intervals (ICI) to adapt the length of the interpolation kernels to the data in order to avoid artifacts. Other examples are [7], where Menon et al. fuse horizontal and vertical interpolations according to local estimations of the image gradient or [8], where Buades et al. propose a version of the non-local means algorithm for self-similarity enforcing demosaicing post processing. Some techniques explicitly view the demosaicing problem as an application of linear filters. Demosaicing with linear filters in the frequency domain was explored in [9]–[11]. Wavelet filter banks are essentially computationally efficient arrays of linear filters, so it should not surprise that several CFA interpolation techniques exist that use wavelet filter banks, e.g. [12]–[15] or steerable pyramid approaches such as in [12]. Menon and Calvagno [15] propose a hybrid technique, performing analysis in the wavelet domain, in order to do adaptive demosaicing in the pixel domain. All these methods have in common that they estimate dominant edge directions by looking at preliminary low-pass interpolated luminance, lowering overall reconstruction bandwidth. Because demosaicing is often performed on low-cost, battery-powered devices, there is an ever present need for computationally efficient demosaicing which delivers high visual quality.

In this paper, we present a method that distinguishes itself by doing demosaicing in a computationally efficient way, by directly performing demosaicing in a multi-resolution sense, without preliminary interpolation, while incorporating the necessary features of a high quality demosaicing algorithm: The algorithm is designed to achieve a higher reconstructed signal bandwidth then many existing methods, combined with locally adaptive measures to avoid reconstruction artifacts. The proposed multi-resolution approach is also notable for its potential to be efficiently extended to wavelet-based denoising and deblurring, which could result in a very efficient joint demosaicing and denoising algorithm.

This paper is structured as follows: in Section 1, we explain the frequency domain view of the demosaicing problem, as well as background on state-of-the-art demosaicing algorithm principles. Section 1.3 details the wavelet interpretation of the demosaicing problem, as this is closely related to the proposed dual-tree complex wavelet implementation. An overview of the proposed algorithm is given in Section 3. Section 2 explains the details of the proposed algorithm, in particular we focus on novelties to the wavelet demosaicing scheme: implementation and filter design issues with respect to the dual-tree complex wavelet transform in Section 2.1, details of the local adaptivity of the proposed algorithm, in Section 2.2, and an approach to extend the reconstructed luminance bandwidth, in Section. Section 3 discusses the performance of several aspects of the proposed technique and compares with state-of-the-art algorithms. Finally, Section 3 concludes the paper.

## Materials and Methods

### 1 Prior art: a Background on Demosaicing

#### 1.1 What is mosaicing?

**Figure 2:**
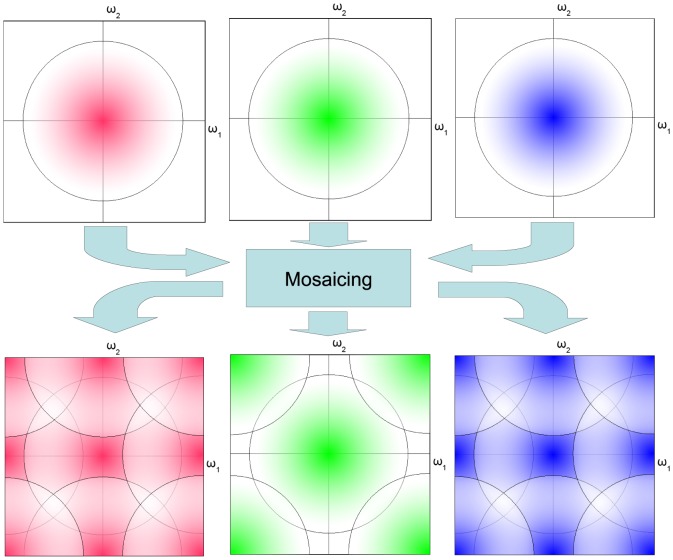
Effects of mosaicing on the signal power spectral density: Three color bands (top row) are subsampled to form one interleaved mosaic image, which is a superposition of the three subsampled color bands (bottom row).

Consider an image which consists of three color channels in an RGB color model. A red channel 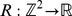, green channel 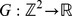 and blue 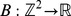. Assuming the Bayer mosaic grid, seen in Figure 1, the mosaic image 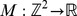 is defined as a superposition of subsampled red, green and blue channels. We use the Bayer grid in this paper, as it remains the most commonly known one, but the principles used in the proposed algorithm can be applied to many different grid layouts, albeit in a heavily modified algorithm: A different sampling grid leads to a different conceptual partitioning of the mosaic spectrum into signal and aliasing. This means that the wavelet analysis and synthesis of color components has to be performed using different equations than the one presented here, perhaps even using more wavelet scales, in order to separate the different parts. However, is still possible to derive a demosaicing algorithm according to the same principles presented in this paper. The mosaicing operation can be interpreted as a subsampling operation. For the Bayer mosaic grid in Figure 1, the image is a sum of three subsampled images, 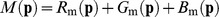, with:
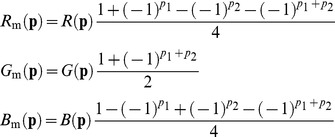
(1)and 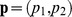 the spatial position. The mosaiced signals discrete time Fourier spectra 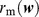, 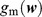 and 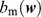 of respectively 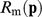, 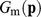 and 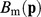 are in fact the spectra 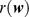, 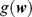 and 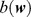 of 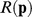,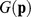 and 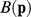 after being subjected to a convolution, which introduces aliasing copies:
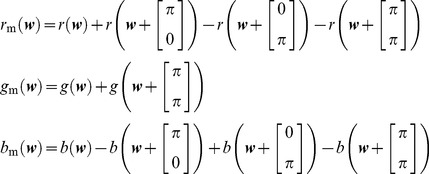
(2)where 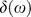 is the Dirac delta function. For visualization and understanding why demosaicing works so well, let us start from a traditional model for natural images: The power spectrum of natural images is decaying with a 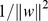 relationship [16], [17]. As such, the power spectrum of the fully sampled respectively, red, green and blue image bands (top row of Figure 2) is visualized as having most of its energy concentrated in the low frequency part. A mosaicing subsampling operation results in interleaved bands which constitute the single band mosaic image. Subsampling introduces aliasing, in accordance with (2), which is visible schematically (by the overlapping circles of equal signal power) in the power spectra of the interleaved color bands (bottom row of Figure 2). Note that the 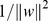 behaviour only holds for ensembles of images, any particular natural image will deviate from this behaviour, significantly so because of sharp lines in the image. Sharp image structures are the cause of demosaicing artifacts in images, will be discussed in detail in the Section 2.2.

#### 1.2 What is demosaicing? The answer using linear filters

**Figure 3:**
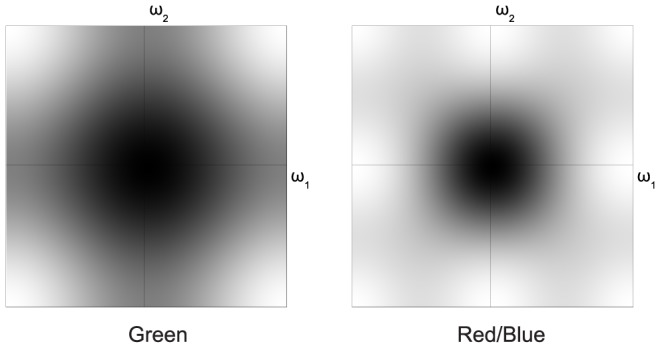
Power spectra of the filters from a bilinear demosaicing filter implementation (black means high power spectral density).

The goal of demosaicing is to reverse the mosaicing operation implemented by the CFA. The most straightforward (linear) demosaicing algorithms demultiplex and filter the different color channels in pixel domain, resulting in a low-pass filtered result. For bilinear interpolation, the corresponding low-pass filters are shown in Figure 3. Note the lower bandwidth for the red/blue filter (right) than for the green filter (left). Also note how the mosaic-related aliases (bottom row of Figure 2) are nicely suppressed by the low-pass filters. The aliasing, related to overlapping power spectra problem has been largely avoided because these low-pass filters have a fairly low bandwidth and essentially serve as aliasing suppression filters. The low bandwidth is a disadvantage, as it reduces image sharpness. The most important way in which more advanced demosaicing techniques, such as the ones mentioned in the introduction, distinguish themselves is by increasing reconstruction bandwidth, while avoiding aliasing/demosaicing artifacts by (simple) non-linear operations. This is also the case for the method presented in this paper. Note that roughly, the bandwidth for green is 75% of the total bandwidth, while the bandwidth for red and blue signals in the reconstructed image is 25% of the total bandwidth.

Many approaches exploit the fact that the human visual system is far more sensitive to luminance, than to chrominance. The Bayer grid was created after this idea and classic color television standards such as PAL exploit this in the modulation scheme [18]. It is possible to exploit this behavior of the human visual system, to increase the potential reconstructed signal bandwidth by making strict bandlimiting assumptions on the chrominance. Where RGB demosaicing assumes low-pass signal behavior on the color channels (as evidenced by the low-pass filters in Figure 3), luminance/chrominance demosaicing uses the assumption of high correlation between the different color channel's high frequencies.

Many demosaicing techniques [2], [3], [5], [10], [14], [19] nowadays incorporate these color correlation assumptions, in one example this is called the smooth hue transition assumption [20]. Another way to achieve similar results, is to approximate luminance as the green value, motivated by the large contribution of the green value to image luma, which is in turn motivated by the human eye's superior sensitivity to greens. The chrominance information is then subsequently considered as the differences red-green and blue-green. This results in a different interpretation of the mosaicing problem:
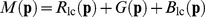
(3)For the Bayer mosaic grid in Figure 1, one can write:
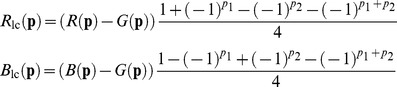
(4)
Figure 1 shows how it is now possible to consider the mosaic image as the superposition of a fully sampled green and two subsampled color difference channels. We will call this the luminance/chrominance interpretation from now. As in Section 1.1, it is possible to translate this demosaicing problem to the Fourier domain interpretation. Note that, when using the luminance/chrominance interpretation, it is no longer possible to perform demosaicing of the three color bands by deinterleaving the subsampled values in the image domain, as the green (luminance) band is not actually fully known at this point, as seen in Figure 1. It could be estimated using a preliminary interpolation step, as is done in many state-of-the-art demosaicing algorithms [2], [6], [12], [13], [15], but we choose not to do so as it increases computational cost. The power spectral density of the mosaic image is shown on the right of Figure 1. Because the bandwidth of the aliased signals (the chrominances or color differences) is assumed smaller for the luminance/chrominance interpretation than for the red/green/blue interpretation from Figure 2, less signal energy is corrupted by aliasing. Hence, it becomes easier to use linear filters to isolate the color bands from their aliases. This explains the power of luminance/chrominance demosaicing: it becomes possible to reconstruct a larger portion of the uncorrupted signal bandwidth than in the RGB demosaicing scenario. If the correlation assumption is correct, it is possible to reconstruct roughly 75% of the total bandwidth for the green as well as the red and blue channels, which is significantly better than in Section sub:Linear-Demosaicing. The difficulty with the luminance/chrominance interpretation is in the demultiplexing of the data. Assuming the combined green signal bandwidth combined with the color differences bandwidth is less than the Nyquist bandwidth, ideal filters (called 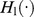 and 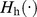 for respectively the low-pass and complementary high-pass filter) can be used to separate the signals from their aliases:
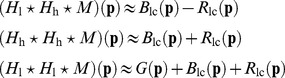
(5)where the signs depend on origin of the Bayer grid. The linear system in (5) can be used to solve for the three color bands 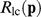, 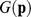 and 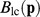, which constitutes the actual demosaicing.

**Figure 4:**
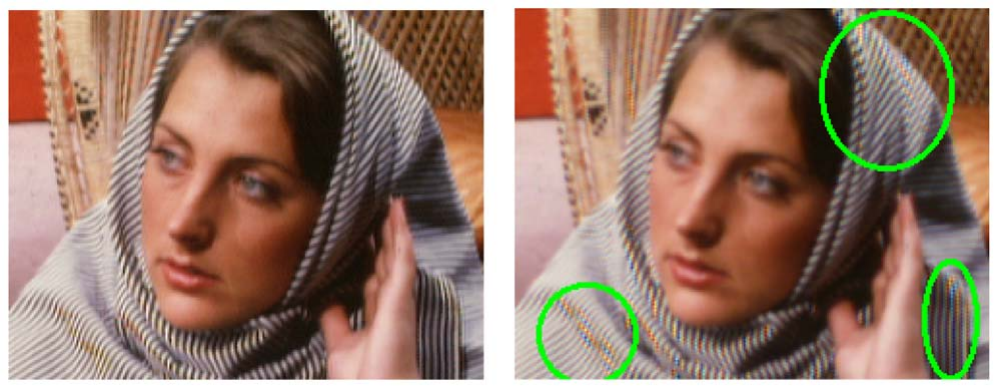
Demonstration of demosaicing artifacts due to local high bandwidth. Bilinear Demosaicing (right) on the Barbara image (original version on the left). note how the local high bandwidth content of the stripes introduces discolorations in the black/white veil, indicated by the highlighted regions.

Natural images still have other characteristics that can be exploited in order to improve demosaicing results. One important characteristic is locality. A natural scene is often composed of different objects so the spectral content of the image normally changes locally across the image. Calculating the global Discrete Fourier Transform (DFT) disallows any local interpretation by averaging any local change in spectral content. As an illustration, we show the result of bilinear demosaicing of the Barbara image in Figure 4. The Barbara image is a public domain test image that is well suited for showing high spectral bandwidth content in images due to the striped cloth and texture. Note the significant demosaicing artifacts on the stripes. The reason is that locally, i.e. if one would only look at the patch of stripes, the power spectral density of these stripes has a very high bandwidth. In light of this, a global set of low-pass demosaicing filters, such as the ones for the bilinear demosaicing in Section 1.1, is a bad choice. Because this significant drawback to global processing, state-of-the-art demosaicing algorithms perform locally adaptive processing in one way or another (such as [2], [5], [7], [19]). Pixel-domain algorithms typically calculate an (sometimes elaborate) edge indicator function, which is then used to fuse multiple directional filter outputs. Several edge indicator functions are used, they are called 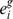 in [19], 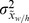 in [2], 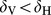 in [7] and 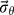 in [5]. In many algorithms, these indicators are used to create convex combinations of different directional estimates [2], [5], [19]. Sometimes, the edge indicators are given a statistical interpretation such as standard deviation for 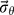 and 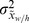 in [5] and [2]. When these are subsequently used in a convex combination, the resulting algorithm implies a Gaussian MMSE estimator of unknown pixel values (in fact, this was formally shown in [2]). In order to keep computational complexity down, we will not use a convex combination to combine directional estimates in our proposed method, but we will rather switch between directional estimates using a statistics-based decision mechanism (explained in Section 2.2). Such decision mechanisms are also used in [7]. For a more thorough explanation of the aforementioned algorithms, we refer to their respective papers. In this paper, we will incorporate the idea of local adaptivity into the wavelet demosaicing framework, which will turn out to be very elegant. For this, we first establish that wavelets can indeed be used for demosaicing.

#### 1.3 What is wavelet demosaicing?

Section 1.1 suggests that demosaicing is in fact an exercise in linear filtering and sampling theory. This approach was taken literally in [11], where Wiener demosaicing filters were designed in the Fourier domain. Section 1.2 suggests that, for natural images, some sort of local adaptivity improves demosaicing results further. Hence, the ideal demosaicing strategy is one that, be that explicitly or implicitly, performs locally adaptive (because of Section 1.2) and frequency selective (because of section 1.2) filtering. Locality is a defining characteristic when comparing the Fourier transform to the wavelet transform. We therefore consider the wavelet transform an excellent choice for demosaicing algorithms. Notably, in [14], such wavelet-based demosaicing scheme is proposed. Demosaicing is achieved through fine tuning of wavelet filters, we will demonstrate this now by modeling the output signal from a single scale wavelet decomposition. We will first explain this concept in 1D, consider therefore the spectrum of a mosaiced, i.e. in this case subsampled, 1D signal:
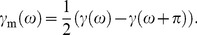
(6)with 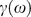 the original signal and 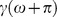 the aliasing copy. Now, assume 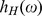 is the frequency response of a wavelet filter. The mosaic signal is filtered and decimated, as in a single scale wavelet transform. The spectrum of the resulting signal is then:
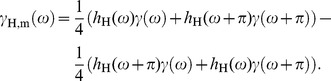
The first term in this equation is the spectrum 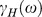 of the wavelet transform of the original signal 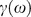. Assuming (approximate) low-pass bandlimitedness of the original signal and the wavelet filter, 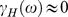. The second term can be rewritten as
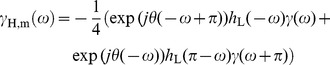
(7)where we assume that the wavelet filters are quadrature mirror filters (QMF), i.e. they satisfy the relationship 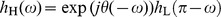, where 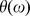 is a phase function, this follows from the definition of a QMF [21]. If the phase function 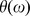 is linear, the phase factor denotes a translation of the signal. Note that 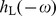 corresponds to a time reversed filter. As such, using a time reversed filter 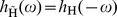 leads to:
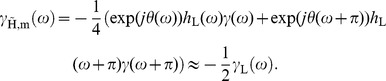
(8)This way, it becomes apparent that the scaling coefficients of a signal can be obtained through filtering the mosaic signal using the time-reversed wavelet filter, but only when the following requirement is fulfilled:
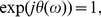
(9)which means that the high pass filter should not be translated with respect to the modulated and time reversed low pass filter. This is an important but not impossible requirement, especially with respect to the design of complex wavelet filters in Section 1.2. Note that it is necessary in order for this technique to work, to use the decimated (i.e. non-redundant) wavelet transform. If the redundant wavelet transform were used, there would be no aliasing term in (7) and as such (8) would not hold. This reduces reconstruction quality as usually the redundant wavelet transform is preferred for restoration purposes because of its translation invariance and associated performance increase with respect to the decimated wavelet transform. This is exactly the problem that we propose to handle using the dual-tree complex wavelet transform.

**Figure 5:**
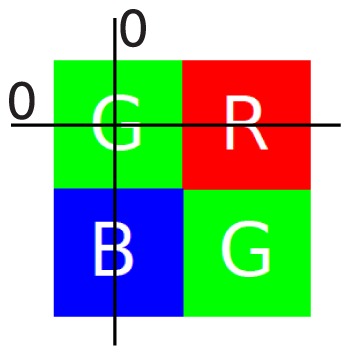
Lattice configuration for the demosaicing procedure.

We now show the concept in two dimensions. Using the relationship (8), one can prove that (for the grid orientation of Figure 4) the following relations hold between the wavelet coefficients 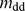 of 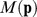 and the wavelet coefficients 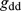, 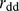 and 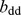 of the target signals 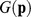, 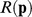 and 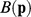, where the subscript 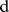 signifies the filter used and we drop the spatial index 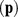 for notational simplicity:
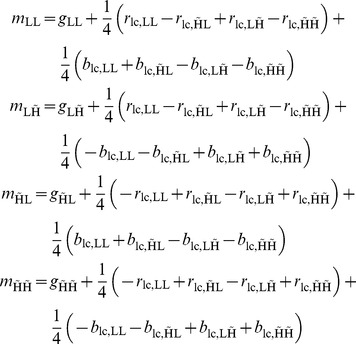
(10)with the subscript 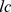 again denoting color differences as in (1). As explained in Section 1.2 and visible in Figure 2, more convenient bandwidth assumptions on the aliased signals can be made by rewriting the signals in a ‘luminance-chrominance’ interpretation. As the color difference signals 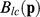 and 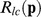 have very small bandwidth, it is reasonable to assume that only their scaling (low-pass) coefficients from a two stage wavelet decomposition will represent significant color difference energy:
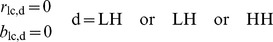
(11)Also in analogy to Section 1.2, the total signal bandwidth, the luminance/green bandwidth added to the chrominance bandwidth, should not exceed the total Nyquist bandwidth. Assuming perfect wavelet filters, this imposes the following assumption on the luminance/green wavelet coefficients:
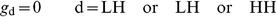
(12)Using (12), (11) and (8), the mosaic wavelet coefficients 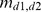 can be expressed as a linear system of equations in the wavelet coefficients of the luminance and chrominance signals 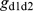, 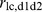 and 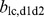;
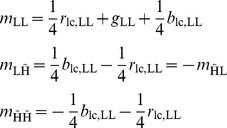
(13)Note that, under the aforementioned assumptions, some coefficients contain the same information
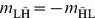
(14)in (13), we will exploit this in the proposed algorithm to perform locally adaptive demosaicing. Resolving this linear system for 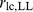, 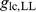, 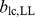 is now well-posed and this solves the hard part of the demosaicing problem: Demultiplexing the three low pass spectral energy components (as seen in Figure 1). This summarizes into the demosaicing rules of Table 1. Using a two level wavelet packet transform, more realistic bandwidth assumptions can be applied to the signal. When attributing 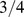 of the bandwidth to green signal and 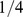 to the color differences, the demosaicing rules in Table 2 are derived. The final demosaiced image can be recovered by using the inverse wavelet packet transform on the demosaiced wavelet subbands of the respective color bands. This demosaicing approach was first proposed in [14]. Note that the demosaicing rules in (2) are only valid for the lattice configuration of Figure 5. Other lattice configurations can easily be handled by using boundary extensions of the mosaic image, or by deriving the analogous demosaicing equations.

**Table 1:** Single scale wavelet subband demosaicing: the three color bands 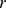, 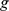, 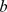
's wavelet subbands in terms of wavelet subbands of the mosaic data 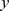.

	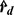	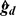	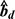
if 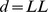	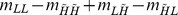	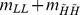	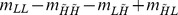
else	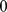	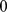	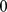

**Table 2:** Two scale wavelet packet subband demosaicing: the three color bands 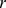, 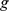, 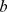
's wavelet subbands in terms of wavelet subbands of the mosaic data 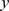.

	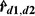	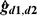	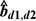
if 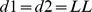	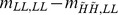	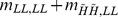	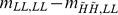
	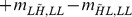		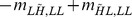
if 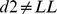	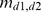	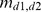	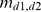
else	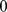	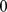	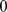

### 2 The Proposed Method

The aim of this paper is now to incorporate the ideas explained in Section 1, such as locally adaptive processing, into the wavelet-based demosaicing framework explained in Section 1.3. We will show that this leads to a novel algorithm that combines the computational simplicity of the wavelet demosaicing framework with the high demosaicing quality characteristics of more complex pixel-domain techniques. We call the wavelet demosaicing framework computationally simple, because the resulting equations, such as (2), are computationally simple. A drawback however, is that in order to obtain alias-free demosaicing, one needs the undecimated wavelet (packet) transforms, which increase redundancy, and with it the computational cost. Another drawback, as described at the end of Section 1.3, is that the derivation of the simple demosaicing equations actually relies on aliasing (2) being present. Instead, we propose the complex wavelet transform as a way to reduce redundancy while maintaining the alias-free processing.

#### 2.1 Dual-tree Complex Wavelet Transform

The dual-tree complex wavelet transform (DT-CWT), originally conceived by Kingsbury [22], introduces several important advantages over the discrete wavelet transform. One advantage is that it allows for shift invariant processing of wavelet coefficients at a lower redundancy than the undecimated discrete wavelet transform. Shift invariance has been long known to be of great benefit for image restoration purposes and in the end demosaicing is image restoration. At the same time, it allows for aliasing in the coefficients, which is necessary for the elegant wavelet domain demosaicing equations in Section 1.3. Because of these characteristics, we find it an excellent choice for wavelet demosaicing.

For a two level wavelet packet transform, the redundancy of the DT-CWT is 4, while for the undecimated discrete wavelet packet transform it would be 16. The aim in dual-tree complex wavelet filter design is to have complex wavelet filters with (approximately) analytic filter responses. To achieve this, the filters and their respective Hilbert transforms are used. The details can be found in [23]. Analytical filter responses are the reason for shift invariance, because shift invariance implies the absence of aliasing in the reconstruction. When using the decimated wavelet transform, shift invariance is lost when processing wavelet coefficients, as the unaltered wavelet coefficients are needed to prevent aliasing, which is present in the scaling coefficients, from propagating into the result. Since the intention of wavelet image processing is to process wavelet coefficients, a different approach to cancel aliasing is often desired. The Hilbert transformed filter bank is one solution. This can easily be seen in 1D, if a signal 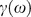 is filtered with a wavelet filter 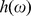 and subsequently decimated, the spectrum of the resulting wavelet band 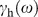 is then:
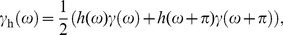
(15)which consists of a correct filter response term and an aliasing term. When doing the same with a Hilbert transformed filter 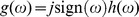, with 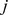 the imaginary unit, the spectrum of the resulting wavelet band 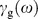 is:
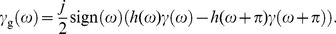
(16)Aliasing, i.e. the 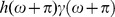 term, can now be demultiplexed by combining (15) and (16), instead of relying on the balance between scaling and wavelet coefficients, as long as the coefficients in the different complex wavelet trees are handled in a way that does not alter the relation between (15) and (16) (e.g. dissimilar scaling or shrinking). This use of the dual-tree complex wavelet transform reduces the redundancy factor of 16 (for a two level undecimated discrete wavelet packet transform) to 4, while maintaining shift invariance and allowing demosaicing. In this work, the mosaic image (3) will be analyzed using a two scale 2D dual-tree complex wavelet packet transform, which has four filter trees. As such, we will denote the mosaic image wavelet coefficients as 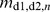, with 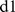 denoting the horizontal and vertical filter pair for the first scale, 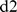 denoting the horizontal and vertical filter pair for the second scale and 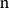 indicating the dual-tree complex wavelet tree, the order of this numbering is of no consequence for this technique. We drop the spatial location index of the wavelet coefficient as our proposed method is fully parallellizable with respect to the spatial location of the wavelet coefficients.

It turns out that analyticity and compact support are incompatible goals. This is why practical implementations settle for nearly analytical wavelet filter pairs. In [23], [24], it was proven that, in order to achieve this (near) analyticity in a filter bank scheme, the first scale wavelet filter 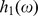 should be shifted approximately one sample with respect to the corresponding filter in the other filter tree:
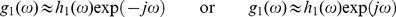
(17)The second and subsequent scales should then use filters 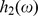 and 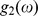 that involve an approximate half sample shift between trees [23], [24]:
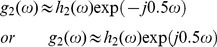
(18)For our demosaicing application, we propose a multiscale (i.e. more than one) complex wavelet packet decomposition, in order to have a sufficiently accurate frequency selectivity as in Section 1.3. Here, we notice a hazard when comparing this with the demosaicing requirements (9): for demosaicing, no shift is allowed between the low pass filter and the time-reversed and (modulated) high pass filter. More formally: if for the filter 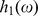 requirement (9) is fulfilled then:
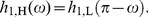
(19)The problem is that (17) actually requires a one sample shift between between the first scale first tree filters 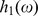 and their corresponding second tree filters 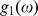 such that:
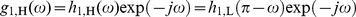
(20)In order to reconcile the requirement that there should be no shift between the low pass filter and the time-reversed high pass filter with the requirement that there should be a one sample shift between the first scale filters in the filter trees, we propose to define the low pass filter for the second tree as:
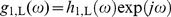
(21)i.e. a shift for the low pass second tree filter in the opposite direction as the high pass second tree filter. Plugging this definition in eq:shifteq, results in:
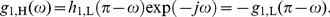
(22)This result shows that the (near) analyticity properties of the dual-tree complex wavelet transform can be coupled with the demosaicing requirement (9). However, a new problem arises: there is now a sign change in the first tree with respect to the second tree, as the filters are designed such that: 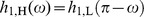 and on the other hand 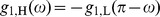. It is very important to account for the sign change in the demosaicing equations, as this will lead to a similar sign change in the wavelet coefficients. It can be compensated for, by changing the signs of the wavelet coefficients with an appropriate factor for every subband in every tree, subsequently applying the demosaicing rules, and then undoing this operation before reconstruction. To avoid confusion with respect to the sign choice, in this paper, we will integrate the sign change into the demosaicing rules, which results in the proposed algorithm having different demosaicing rules for the different filter trees, which we will explain further.

#### 2.2 Locally adaptive demosaicing using (complex) wavelets

**Figure 6:**
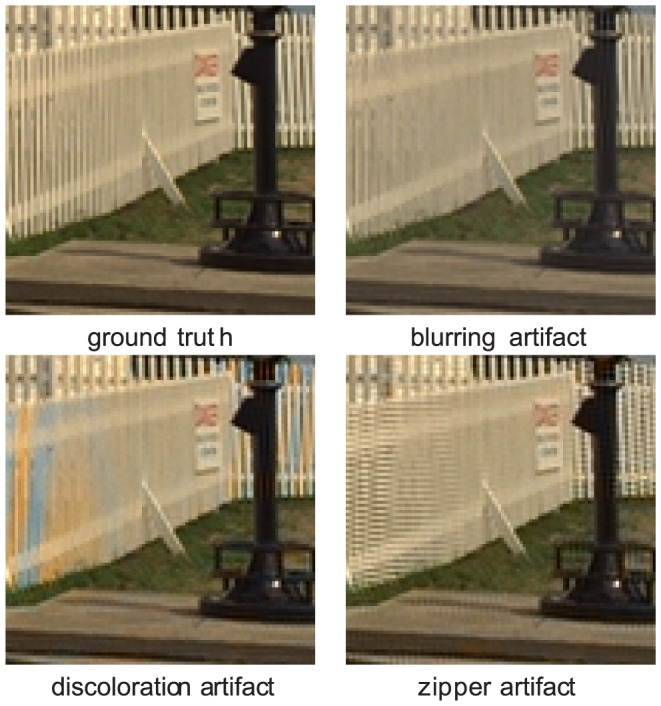
Demosaicing artifacts examples.

As described in Section 1.2, spatially invariant demosaicing algorithms, such as bilinear demosaicing algorithm, fail in regions with high spatial frequency content (e.g. the shroud of Barbara in figure 4). One could say that the implicit bandwidth assumption, i.e. that the signal is bandlimited to a low-pass behavior, is locally invalid. As a spatially invariant algorithm, the wavelet demosaicing approach described in Section 1.3 suffers from similar problems. We will focus on the following artifacts:

- Only 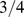 of the maximum luminance bandwidth is reconstructed. This means that the remaining 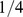 of the luminance bandwidth is not reconstructed. This lack of bandwidth leads to blurring with respect to the reference image. This can be seen in Figure 6.
- Luminance energy beyond the 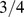 bandwidth point is confused for chrominance energy, which results in severe discoloration artifacts in the result. This can be seen in Figure 6 (second from right).
- Incorrect detection and processing of a local feature leads to incorrect new edges, which we call the zipper artifact. This can be seen in Figure 6 (right).

**Figure 7:**
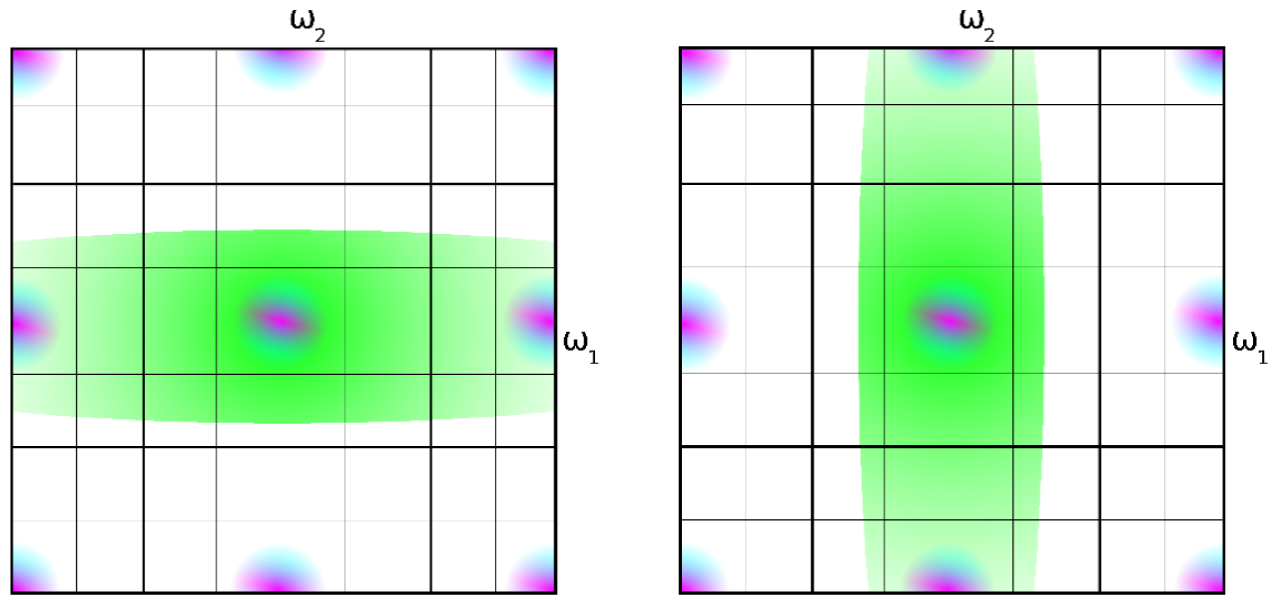
Color corruption can be caused either by excess luminance bandwidth in the vertical or horizontal direction.

Other artifacts relate to the low bandwidth reconstruction of chroma, 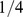 of the total bandwidth. As these artifacts are psycho-visually not disturbing, which is strongly related to the efficacy of chroma subsampling in compression schemes [25], we will not take special measures to correct them. Eliminating the aforementioned artifacts consists of two steps: detecting demosaicing artifacts (Section 2.2) and correcting them (Section 2.2 and Section 2.2). The strategy to correct these artifacts is based on the redundant information in the demosaicing equations (14), which is in turn related to the existence of multiple aliasing copies of the chrominance signals, as they are subsampled both vertically and horizontally in the mosaicing process. If two uncorrupted aliasing copies of the chrominance signals can be found in the spectral content of any of the mosaic (complex) wavelet coefficients, artifact-free reconstruction is possible. Consider an image patch with some vertical stripes, i.e. large horizontal bandwidth (e.g. the picture in Figure 6). Now the luminance bandwidth assumption (12) is no longer correct. For our two level wavelet packet transformation we can express this as:
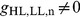
(23)As a result, the simplified equations from which the demosaicing rules are derived (13) are also incorrect, and instead become, for the first of the four trees of our two level 2D wavelet packet transformation:
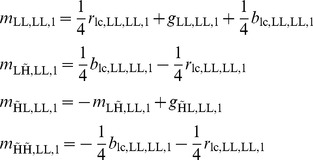
(24)The wavelet coefficient 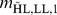, which should only contain chrominance alias, now contains excess luminance energy and is considered corrupted. In a demosaicing algorithm that is oblivious to these artifacts, such as when applying the algorithm in Table 2 separately to the different complex wavelet trees, the result would show a discoloration artifact. The red, as well as the blue, low-pass signal is corrupted:
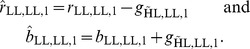
It is equally possible that the artifact was caused by horizontal stripes, i.e. large vertical bandwidth. Before an attempt can be made to suppress the artifact, it should be known whether the artifact is caused by excess luminance bandwidth in the horizontal direction (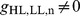, graphically in Figure 7, left) or by excess luminance bandwidth in the vertical direction (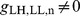, graphically in Figure 7, right). In theory, it could happen that the diagonal wavelet coefficient is corrupted (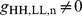), but since this represents a higher bandwidth than the vertical and the horizontal wavelet coefficient, we do not consider this here.

**Figure 8:**
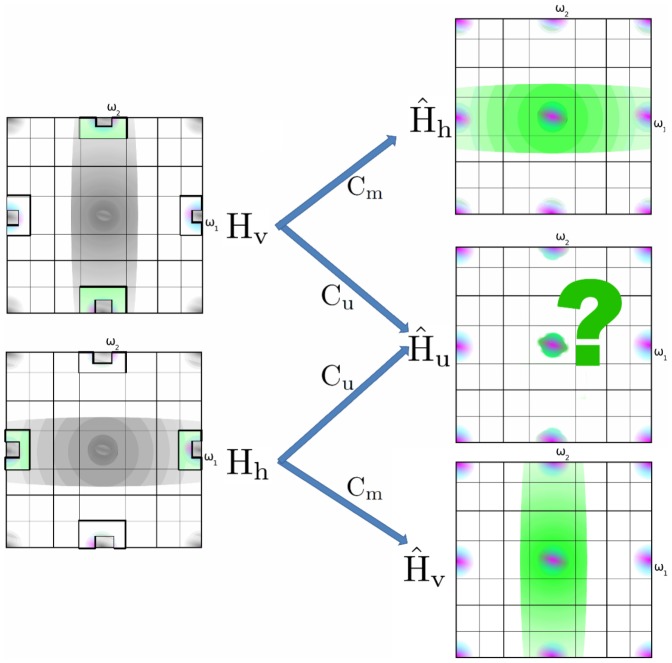
From the filtered wavelet coefficients 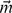, whose support is indicated by the colored area on the left, and two initial hypotheses: either high horizontal luminance bandwidth (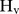) or high vertical luminance bandwidth (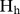) is dominant, the costs of making an “incorrect” decision 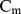 or an “unsure” decision 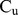 is indicated. The cost for a “correct” decision 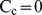 is not indicated.

**Figure 9:**
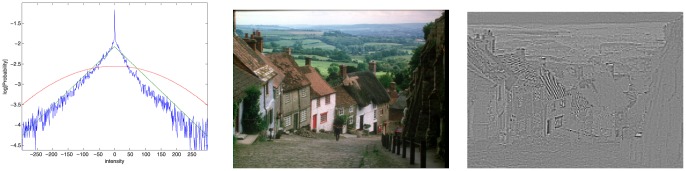
Demonstration of the suitability of a Laplacian model on the high pass band 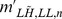. Original image (middle) and its high pass band 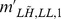 (right) and its logarithmic histogram of these coefficients (left), along with a Laplacian distribution fit (green) and Gaussian fit (red).

We will now look at detecting demosaicing artifacts. In the previous section, it was remarked that, when there are no artifacts, 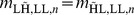. When artifacts occur, these coefficients are corrupted with an extra luminance term, such as in (24). The aim now is to detect which of these coefficients is the least corrupted. Since the visual quality of locally adaptive processing is sensitive to incorrect detections (see the zipper artifact in Figure 6), we will develop a Bayesian *multihypothesis* technique to decide which one of both coefficients is corrupted. There are two hypotheses: 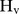, which means that there is a dominant local horizontal feature (i.e. locally larger vertical luminance bandwidth) and 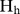, which means a local vertical feature. Based on those two starting hypotheses, there are three possible decisions: 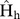, which means a vertical feature is detected; 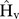, which means a horizontal feature is detected and 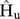, which means either of the previous decisions is too dangerous with respect to the cost function (this is the “unsure” decision). By introducing this third hypothesis, we can avoid visual artifacts (Figure 6) that would otherwise originate from incorrect detection of 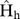 or 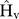. The Bayesian risk that is to be minimized by the decision is:
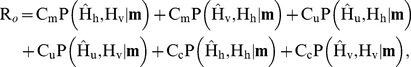
(25)where 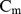 is the cost of an incorrect decision (a “miss”), 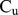 is the cost of an “unsure” decision and 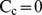, the cost of a “correct” decision. Instead of basing the decision on the vector 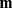 of all wavelet coefficients associated with a given spatial location, we base the decision on a corruption measure. In order to distinguish the (luminance) corruption from the chrominance in the analysis, we propose the use of a third level in the wavelet packet decomposition, which can be performed at no extra memory cost using the dual-tree complex wavelet transform. We apply 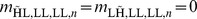. Reconstruction of this filtered third scale will lead to a approximate chroma-free coefficients, which we define as 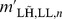 and 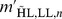. Note how this zero setting operation acts as a simple band reject filter, where the dual-tree complex wavelet transform provides an efficient way to implement it. These coefficients are subsequently used as the corruption measure, i.e. an estimate for high frequency luminance. In this framework, the decision is made that minimizes the risk (26), given the corruption measure:
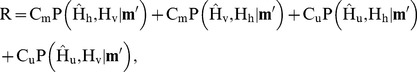
(26)with 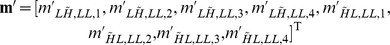 the observed vector of filtered coefficients at a given spatial location in the four complex wavelet trees. The hypotheses and associated cost for a “miss” decision 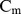 or an “unsure” decision 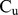 are visualized in Figure 8. Assuming 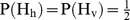, which means that horizontal edges and vertical edges are equally probable, (26) can be expanded into
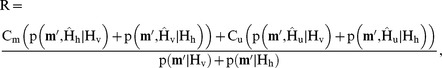
which can be further simplified into
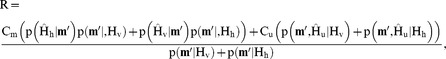
where we make use of the fact that the decision only depends on the measurement vector such that
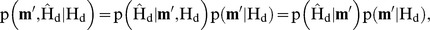
with 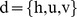. Now, we note that the decision is deterministic such that the functions 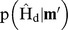 are binary. The minimizer of this risk in this setting is shown in (27).
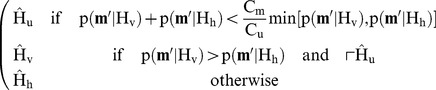
(27)We now propose a Laplacian model for the statistics of filtered wavelet coefficients 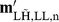 and 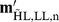 :
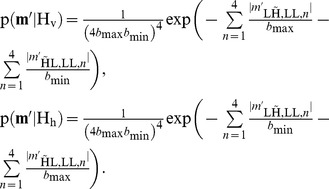
The motivation for a Laplacian model lies in the highly leptokurtic statistics of bandpass filter output when dealing with natural images. This is well known in image restoration [26]–[30]. For the specific case here, on a filtered dual-tree complex wavelet packet transform band, we illustrate the validity of this using statistics extracted from the goldhill image, shown in Figure 9. The figure shows a logarithmically plot histogram of coefficients 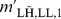 from a natural image, along with a Laplacian fit, and a Gaussian fit. It can be seen that the Laplacian fit is indeed very accurate. The parameters 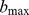 and 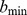, which are related to the variance in respectively the dominant and the subordinate direction remain to be estimated. Since we have 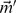 available, and our initial hypothesis model assumes a dominant direction in all scenarios, we estimate these from the sample coefficients in both the horizontal and vertical direction in an maximum likelihood sense. The largest of these two estimates is then used as a maximum likelihood estimate for the parameter 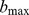, the smallest for the parameter 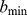:
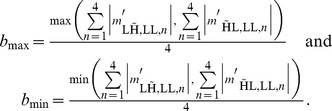
For computational simplicity we assume that the four coefficients 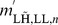, as well as the four coefficients 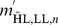, are conditionally independent on the initial hypothesis. This is an effective simplification: the choice for a single 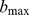, respectively 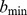 parameter for the coefficients in a single filter direction reflects the filters in the four complex wavelet trees having nearly the same magnitude response. The choice for a model without correlations between the coefficients is motivated by the significant phase shift between coefficients in the different trees and computational simplicity. Using this Laplacian model, this decision rule can be effectively simplified:
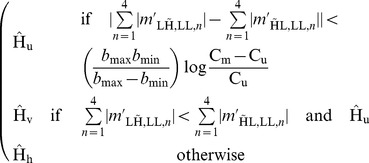
(28)The costs 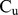 and 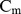 are chosen to minimize reconstruction error, which is explained in the Section.

**Figure 10:**
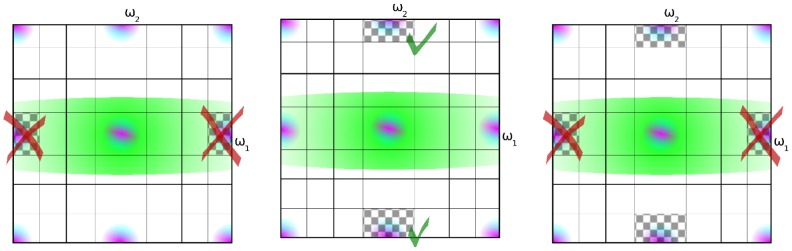
The detection framework choses one hypothesis, from left to right 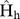, 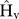 or 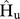, and uses a corresponding reconstruction rule (the checkered chrominance alias). Only one hypothesis uses exclusively uncorrupted chrominance.

**Figure 11:**
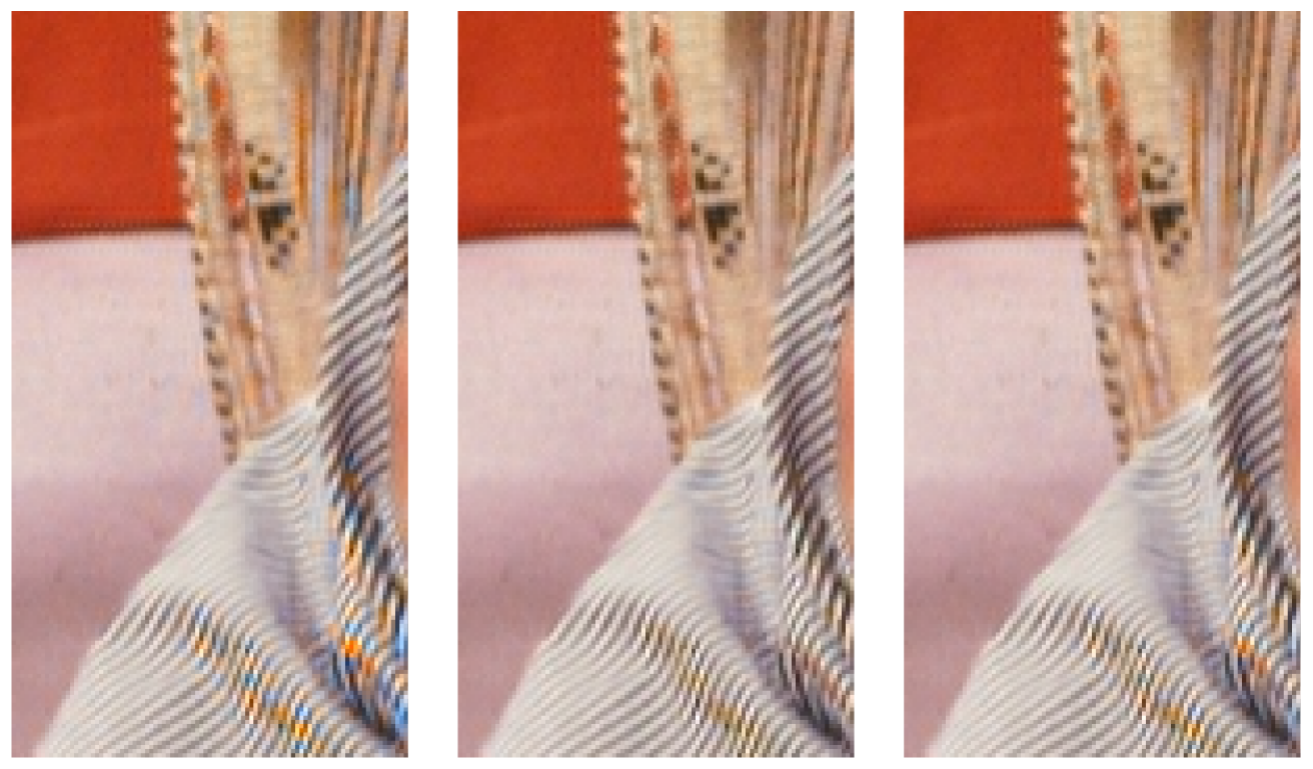
Demosaicing of Barbara image for the three demosaicing rules in figure 10. The incorrect rules (left and right), which use corrupted aliases to reconstruct chrominance, lead to local discolorations near high frequency regions, whereas the correct rule (center) results in no discolorations.

Now that we now know how to detect demosaicing hazards, artifact-free reconstruction is easy. Locally, it is now possible to detect which of the chrominance coefficients 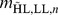 or 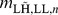 are uncorrupted by luminance and then subsequently use these in the reconstruction, as in Figure 10. Using the (complex dual-tree) wavelet packet transform, this local demosaicing can be implemented, precisely because of the aforementioned ambiguity in the demosaicing rules presented in Section 2: under the assumptions of small chrominance bandwidth (12) and small luminance bandwidth (11), it follows from (10) that 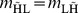. Realizing this, the first line in 2 can be rewritten as in Table 3. Note that these equations are only valid for the first complex wavelet filter tree, as the sign change (22) results in switched signs for some coefficients in these equations, the derivation of these formulas for the other complex wavelet trees is not mentioned here to conserve space, but is completely analogous. Alternatively, the signs could be changed as a preprocessing step. From Figure 10, it is seen that there is only one situation where only uncorrupted bands are used, i.e. only one demosaicing rule will lead to correct colours in the demosaicing result. The consequence of using the different demosaicing rules is depicted by the comparison in Figure 11. Figure 11(left) suffers from the worst colour distortions, which is explained through the use of only corrupted chrominance aliases (Figure 10(left)). Conversely, Figure 11(middle) suffers from the least colour distortions. Figure 11(right) represents a kind of middle ground. Here the corrupted chrominance information of the 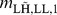 subband is mixed with the uncorrupted information of the 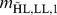 band. The global wavelet-based demosaicing approach, the one originally used in [14], corresponds to the middle ground concerning demosaicing artifacts (Figure 11c) by averaging the uncorrupted with the corrupted coefficient. In our proposed locally adaptive complex wavelet-based demosaicing, we switch locally between the demosaicing rules in Table 2 and Table 3, depending on the detection result explained in Section 2.2. On top of that, this approach maintains the translation invariance so far as possible, as the detection result (28) is constant across the different complex wavelet trees. Using (28), the cost of making a “miss” decision 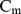 can be weighed against the cost of making an “unsure” decision 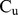. Let 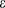 be the erroneous contribution due to luminance in one of the chrominance coefficients. The accumulated errors in the reconstructed coefficients can easily be calculated using (5), they are shown in Table 4.

**Table 3:** Alternatives for the low pass wavelet demosaicing rules when compared to Table 2.

	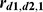	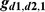	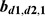
if 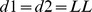	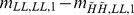	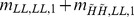	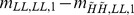
	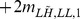		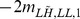

**Table 4:** Comparison between the errors accumulated in the low pass coefficient, due to either a “miss” decision and an “unsure” decision.

	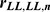	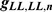	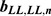
“miss” decision	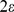	0	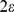
“unsure” decision	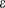	0	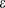

**Figure 12:**
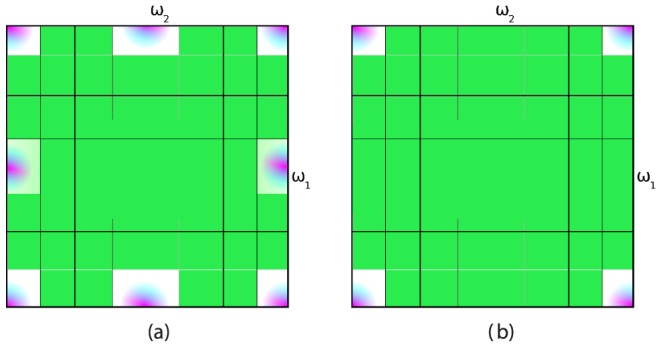
Comparison of reconstruction bandwidths when extended demosaicing rules are used. Reconstructed luminance bandwidth (indicated by the spectral support in green) of (a) the reconstruction rules in Table 5 and (b) the reconstruction rules in Table 5 combined with the rules in Table 6.

The reconstruction rules in Table 5 allow for a reconstruction of the luminance bandwidth that is limited to the regions indicated in green in Figure 12(a). We now investigate the possibility of extending the reconstruction bandwidth for the luminance to the one shown in Figure 12(b). We take a look at the wavelet coefficients 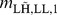 and 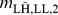 under hypothesis 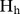, which means a dominant horizontal local feature. In this scenario, we may write:
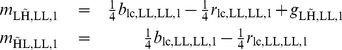
(29)This opens the possibility of reconstructing the high frequency luminance as 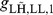 = 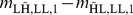. Similarly, for hypothesis 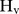, it is possible to reconstruct 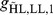 = 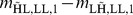. Reconstructing these high luminance frequency coefficients this way is undesirable, as requires compensating for the time inverted wavelet filter 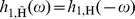 in the reconstruction, increasing the complexity, however we note that this can not be avoided: Consider filtering the mosaic with non time inverted first scale wavelet filters, then exploiting that (9) holds such that 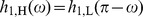:
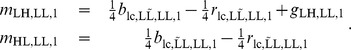
In this case, 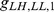 = 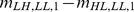 only holds when
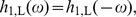
i.e. when the lowpass filter is a perfectly symmetric filter, which is usually not the case. Still, symmetric filters can be implemented for the first complex wavelet tree, but it becomes problematic when looking at different trees of our dual-tree complex wavelet packet decomposition. This is because of the one sample shift requirement for complex wavelet filter trees (21), as now:
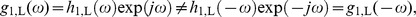
i.e. in anything but the most trivial case (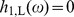), is impossible for both the filters 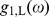 and 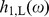 to be perfectly symmetric. This makes it impossible to use the normal reconstruction filter bank when reconstructing these high frequency luminance coefficients. We therefore revert to the first idea, to use the reconstruction rules in Table 6, which in contrast with the rules in Table 5 make use of time reversed wavelet reconstruction filters, which increases the implementation complexity. Again, we take a look at the errors accumulating when the wrong hypothesis is chosen, these are shown in Table 7. Since the dual-tree complex wavelet transform is a Parseval frame, the influence of the coefficients in Table 4 and Table 7 can be directly related to mean square error (MSE) in the image domain. The MSE due to a “miss” decision is, which we will use as the cost in (28) is 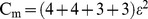, while the cost for an “unsure” decision is 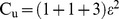. This leads to a ratio 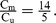 The significance of this result is that it pays off to include the “unsure” decision in our decision framework as it can be seen from (28) that the decision 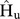 will only improve MSE and be used when 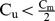, which is true in this case.

**Table 5:** Locally adaptive complex wavelet subband demosaicing for the first tree: the three color bands 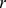, 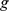, 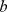
's wavelet subbands in terms of wavelet subbands of the mosaic data 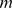.

	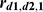	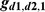	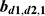
if 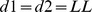	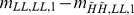	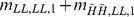	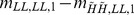
and 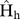	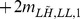		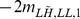
elseif 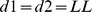	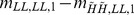	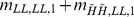	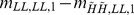
and 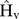	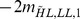		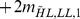
elseif 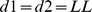	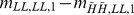	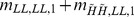	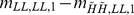
and 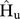	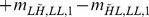		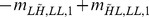
if 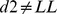	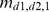	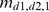	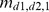
else	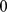	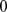	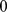

**Table 6:** Demosaicing rules for the extended luminance bandwidth coefficients beyond the ones in table 5, for the first tree.

	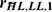 , 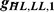 , 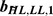	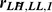 , 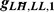 , 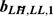
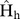	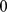	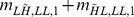
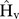	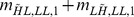	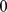
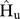	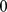	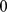

Note that the inverted reconstruction filters need to be used here.

**Table 7:** Comparison between the errors accumulated in the high pass coefficient, due to either a “miss” decision and an “unsure” decision.

	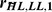 , 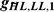 , 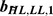		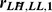 , 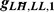 , 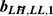	
	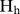	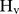	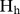	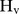
“miss” decision	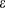	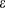	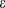	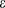
“unsure” decision	0	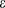	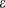	0

### 3 Overview of the proposed algorithm

**Figure 13:**
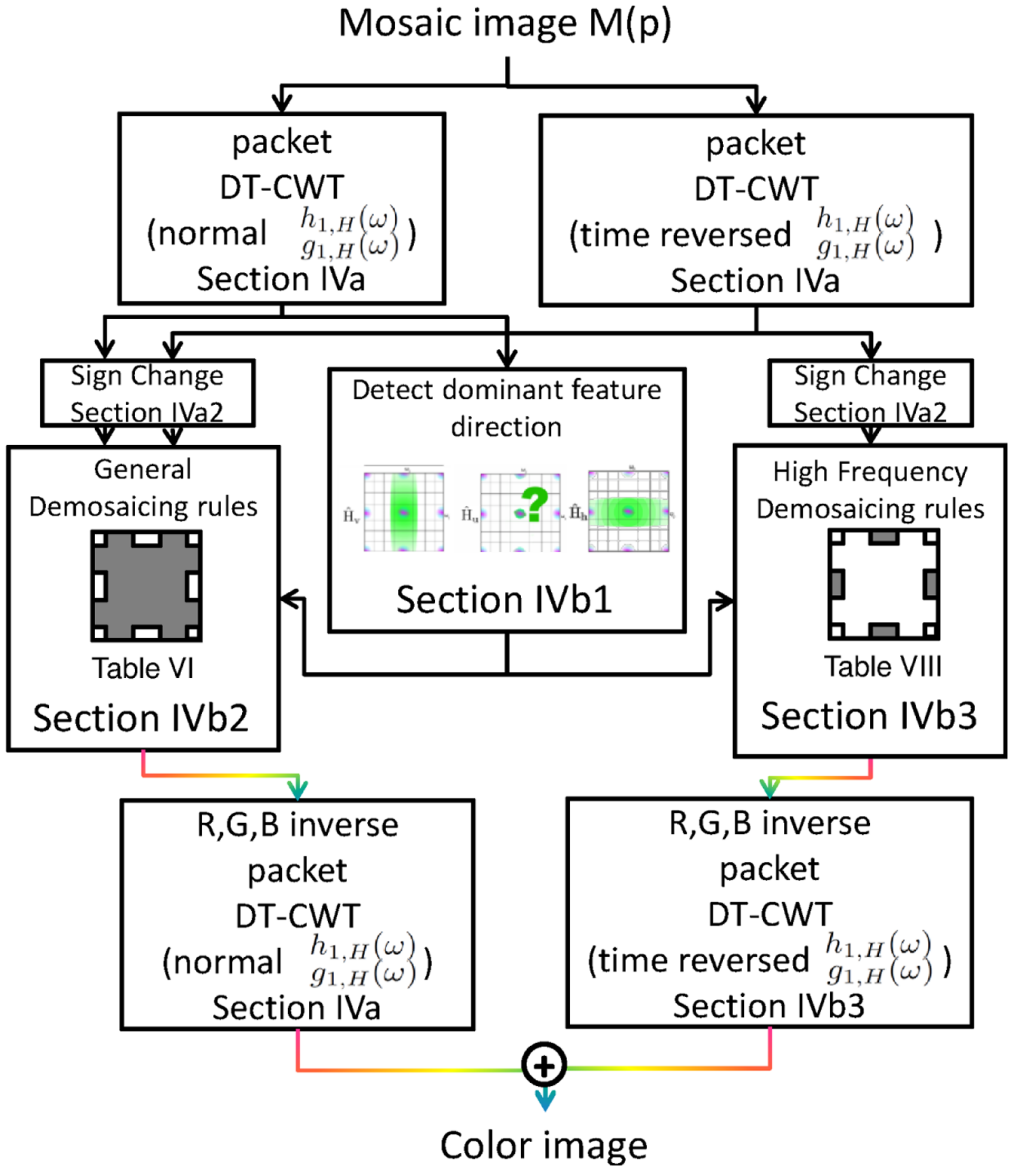
Flowchart of the demosaicing algorithm. The greyed area shows which part of the R,G,B spectrum is recovered in each step, for each tree. The relevant sections for each block are mentioned.

**Figure 14:**
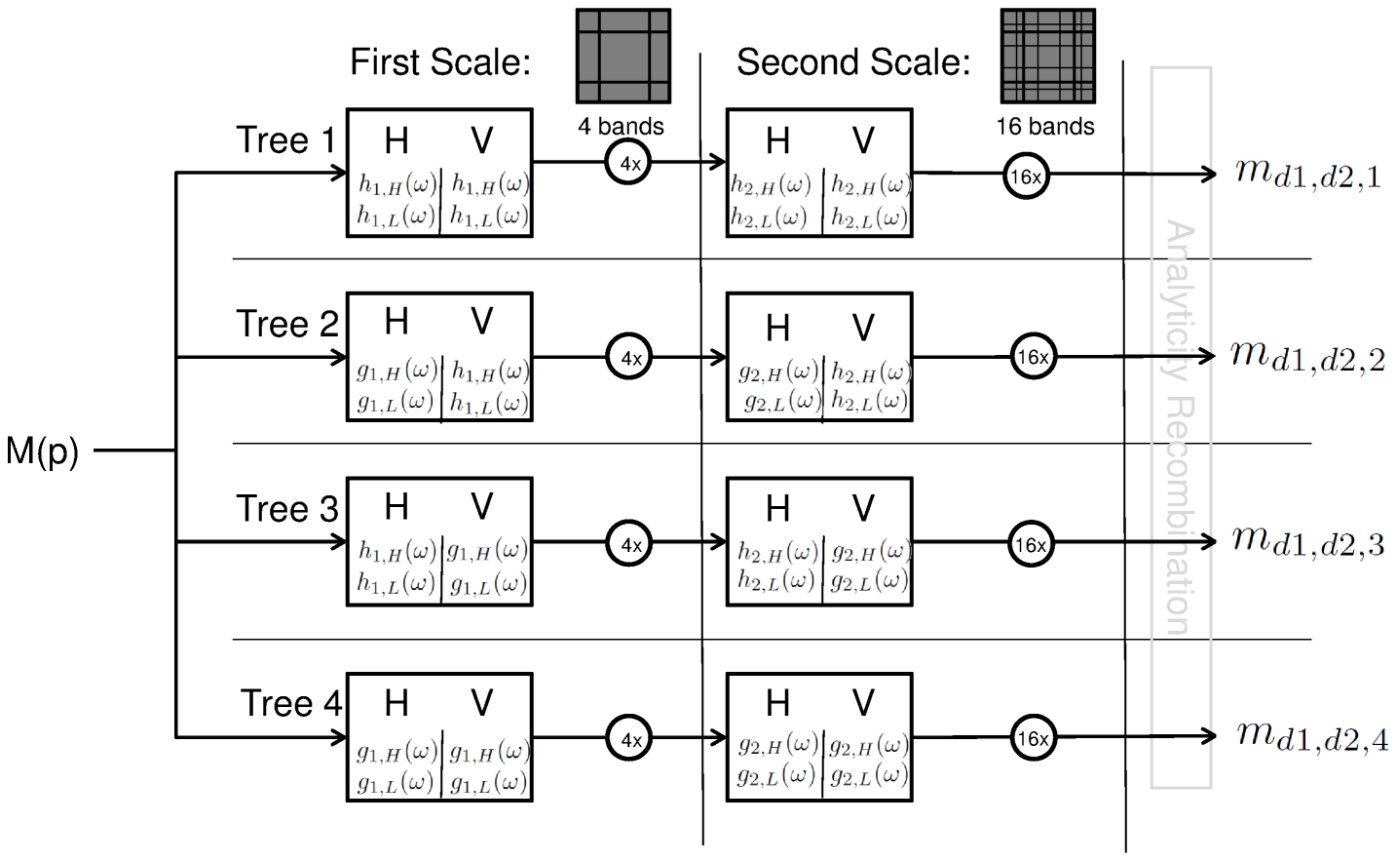
Flowchart for the two scale dual-tree complex wavelet transform as used in this paper. Note that the analyticity recombination, see text, is not performed for in this paper.

A complete flowchart of the algorithm is shown in Figure 13. Without loss of generality, the assumed top left of the input Bayer mosaic is oriented as in Figure 5. Conceptually, there two concurrent dual-tree complex wavelet packet transformations needed for a basic implementation, such as the one implemented for this paper. However, computational complexity can be reduced due to the large number of zero, unused wavelet bands. Memory complexity can be halved as the non-zero complex wavelet bands in both transforms are mutually exclusive (compare the bands set to zero in Table 5 with Table 6). The first scale filters need to be designed according to requirement (9), i.e.
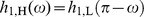
The first scale filter for the second complex wavelet tree should then be chosen according to requirement (21) and (20):
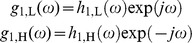
For the second scale of the complex wavelet filters there are no further special requirements concerning this demosaicing application. The need for a different demosaicing procedure for every complex wavelet tree has its origin in the sign change which is introduced into the demosaicing equation by the Hilbert transform first scale filter (22). The flowchart for the DT-CWT transforms is shown in Figure 14. For a 2D DT-CWT, it is necessary to perform a linear transformation of the output of the four separate filter trees, because only then the coefficients have an interpretation as coefficients of a complex 2D wavelet (see section “2-D dual-tree CWT” in [23]) and only then (diagonal) directional analysis is possible. We call this a recombination step. For example, from [23] we get that the real part of a 2D complex wavelet can be obtained as:
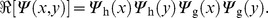
For this work, this means subtracting wavelet bands such that:
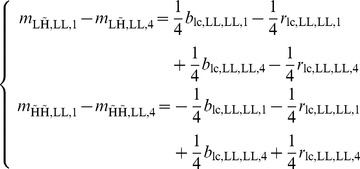
We forgo this recombination step in this paper, as this step complicates derivation of the demosaicing rules and in the proposed method no diagonal analysis is used. However for future work, we remark that diagonal directional analysis could open up the possibility of reconstructing even more of the original luminance bandwidth in the diagonal direction, at the cost of added implementation complexity.

## Results and Discussion

**Figure 15:**
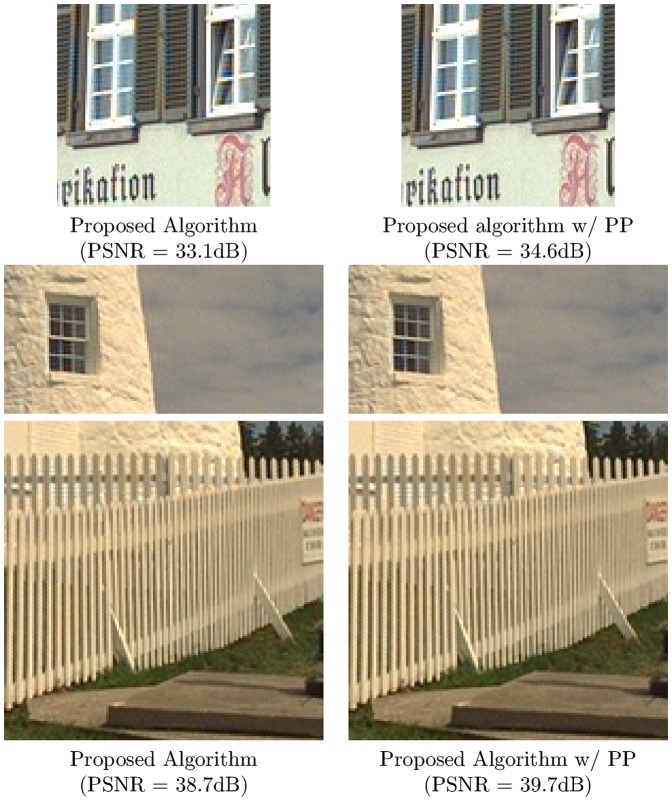
Effect of postprocessing on the proposed demosaicing algorithm. Note the negligible visual difference, but the large difference in PSNR due to the data fidelity property.

**Figure 16:**
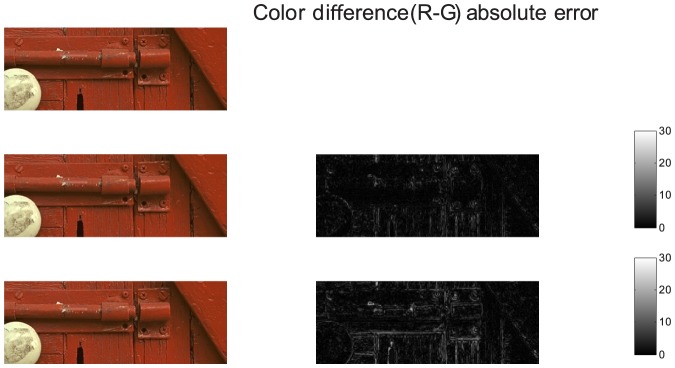
Demonstration of the artifacts occuring with high chrominance bandwidths. Top: Ground truth image, Middle: LPA-ICI (PSNR = 41 dB), Bottom: Proposed Algorithm (PSNR = 39 dB).

**Figure 17:**
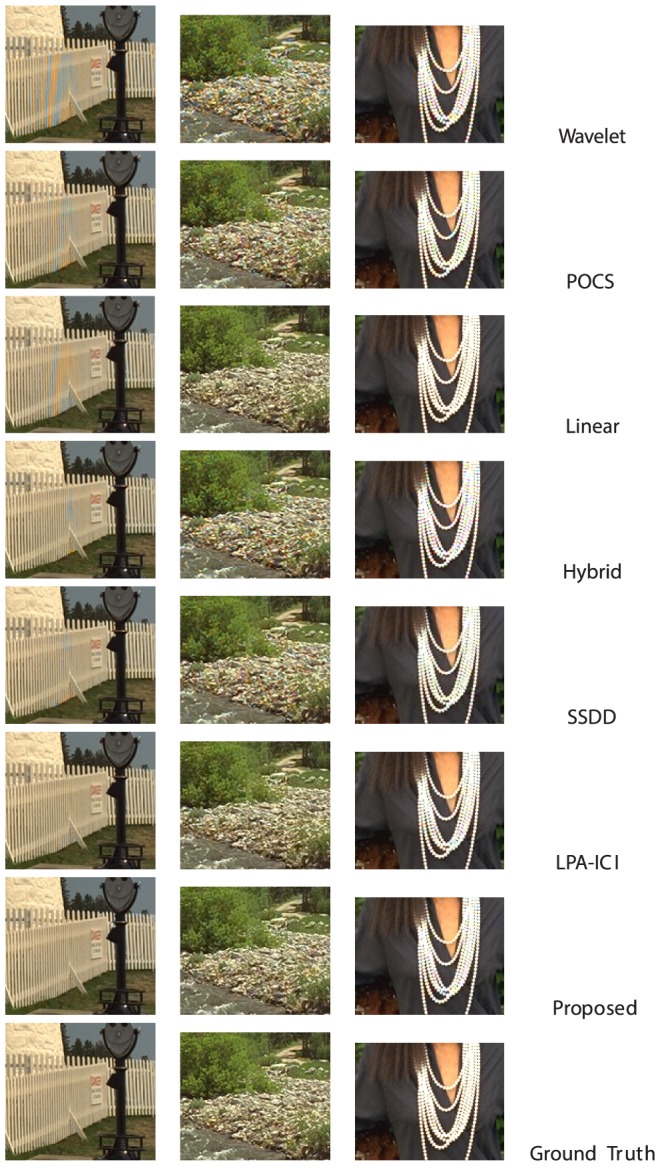
Demonstration of the high luminance bandwidth reconstruction properties, note the blue and orange artifacts due to excess luminance bandwidth.

In this section, we compare the demosaicing performance of the proposed algorithm with several other algorithms. In our comparison we will use the non-adaptive wavelet demosaicing algorithm from [14], the DLMMSE method from [2], the POCS method from [3] (set to a fixed number of 5 iterations), the hybrid [15] (wavelet detection and pixel based reconstruction), the linear filter scheme from [10] and [5], which is the qualitative state-of-the-art at the moment of writing, to the knowledge of the author. The implementations used here are publicly available from http://www.csee.wvu.edu/xinl/source.html, except for [10] which we implemented based on the suggested filters in [10]. While the proposed approach for demosaicing has significant advantages, there is a drawback when it comes to objective comparison. The algorithm makes hard assumptions on the chrominance bandwidth, any small error thus introduced in a wavelet coefficient will cause a hue shift across all pixels in the wavelet's support. Upon visual inspection, these small errors are hardly noticeable, but they can result in a significant MSE. In order to decrease the MSE, we could simply insert the measured pixel intensities from the mosaic into the reconstructed image, however, this gives rise to very noticeable zipper artifacts (see for an example Figure 6). This indicates that MSE or PSNR have severe drawbacks as a measure for visual quality. We still choose to use it here, as it remains the most popular choice of comparison in literature. In order to have a fairer comparison with respect to visual quality, we apply the demosaicing post-processing technique from [31]. This algorithm exploits the spectral correlations between the color components and the luminance bandwidth (i.e. sharpness) that was introduced in the proposed demosaicing algorithm to estimate only the missing pixel intensities, starting from the measured pixel intensities and preliminary interpolations. Hence, it retains the artifact-reducing power and high luminance bandwidth advantages of the proposed algorithm, as we will use this as preliminary interpolation, but it increases PSNR. The visual quality is related to the artifact-reduction and, as a result, is not improved. This effect is demonstrated in Figure 15: even though there is an increase of more than 1 dB in PSNR, there is hardly any visual difference, even in these difficult demosaicing experiments. In the remainder of this paper, we will compare PSNR results of the proposed algorithm with post-processing enabled. It is important to note that other demosaicing algorithms (such as [2], [5]) already have data fidelity: They do not modify measured pixel values from the input grid. For these algorithms, as they already have data fidelity, it makes no sense to apply this post-processing and we repeatedly found it only reduces their respective performance. The proposed algorithm was tested on the 24 512×768 images of the classic Kodak test image data set (http://r0k.us/graphics/kodak/). Table 8 shows PSNR comparison for the different algorithms, compared with the proposed algorithm. We also list the results for the proposed algorithm when the dual-tree complex wavelet transform is not used. This demonstrates that the use of complex wavelets has a significant impact on the result with respect to aliasing reduction in the result, on average it means an increase of 1 dB in PSNR. The PSNR comparison shows that the proposed algorithm holds itself quite well, with respect to the state of the art in demosaicing algorithms. It shows how the wavelet-based methods, due to the crude assumptions made on the transition bandwidths, are outperformed by the pure linear filter scheme from [10], which has finer control over the transition bandwidth, when the local adaptivity of wavelets is not exploited. The local adaptivity however, is shown to be a significant improvement over non-adaptive wavelet schemes (e.g. from [14]) as well as the purely linear scheme in [10]. While some of the pixel-based demosaicing algorithms achieve a significantly higher PSNR in some experiments, this is mainly due to chroma shifts in the proposed algorithm. These small shifts are not as visually disturbing as structural demosaicing artifacts, which manifest more often in other algorithms, as large high frequency luminance+chroma errors. To demonstrate this point, we also include a qualitative comparison. Figure 16 shows the chrominance bandwidth problem in the most problematic image, i.e. the one with the highest PSNR difference between LPA-ICI and the proposed algorithm. Here we see a lot of high frequency edges between black areas (low chrominance) and red areas (very high chrominance), hence we have high chrominance bandwidth locally. Here, the low chrominance bandwidth assumption fails, and artifacts are introduced. However, we remark that these artifacts are not as visually disturbing as other demosaicing artifacts, which the proposed algorithm handles very well. One example is the classic lighthouse1 image from the same Kodak dataset. A comparison is shown in Figure 17. All demosaicing algorithms have difficulties reconstructing the white fence and the rocky river bank, because of its very high luminance bandwidth. The proposed algorithm is capable of reconstructing the luminance to the original Nyquist bandwidth, as discussed in Section 2.2, which leads to better reconstruction performance in comparison with other demosaicing algorithms in areas where the luminance exhibits a high bandwidth. A comparison of local PSNR, for these artifact-sensitive regions is shown in Table 9. Another big advantage of the proposed method lies in its computational simplicity. We compared the available Matlab implementations of the different demosaicing algorithms with respect to their average execution times on the 24 images of the Kodak set. The result is shown in Table 10, the proposed algorithm, in its naively implemented state, is significantly faster than the existing state of the art in demosaicing, while achieving a roughly equivalent qualitative, and in some respects (luminance bandwidth) better, demosaicing result. For POCS, we also mention the estimate that takes the speedup (factor 8.5) into account of the accelerated version of the POCS algorithm presented in [4]. The addition in between brackets expresses the time it takes to perform the wavelet transform, we make a distinction here as the wavelet transform can immediately be made use of to perform other restoration tasks than demosaicing, such as denoising, sharpening, etc. Combining wavelet based demosaicing with more general restoration has already been demonstrated in [32], which highlights the relevance of the proposed technique.

**Table 8:** Quantitative demosaicing result (in dB PSNR) for the different demosaicing algorithms on the Kodak data set.

	Bilinear	Proposed	Proposed w/o	Proposed w/o	DLMMSE [2]	Linear [10]	POCS [3]	LPA-ICI [5]	Wavelet [14]	Hybrid [15]
			Post Processing [31]	complex wavelets						
Facade	25.7498	37.3104	35.6445	35.9515	38.4469	35.4060	36.267	40.4238	31.5523	36.5619
Gate	31.509	38.8959	38.2277	37.8592	38.7158	32.6866	37.6156	41.0878	34.2818	38.6346
Caps	32.4473	41.7574	41.3494	40.8284	42.6786	36.2425	39.2148	43.6412	36.1925	42.1746
Red hat	32.1083	40.4016	39.8044	39.7036	37.4983	35.8707	38.8279	40.7697	35.9086	40.2272
Bike	26.0905	37.4384	36.8965	36.6425	38.1296	34.7838	36.4263	37.692	33.4215	36.9755
Fishing	27.1191	39.5872	38.2944	38.0588	39.9935	36.0580	36.7864	40.9305	32.1537	38.538
Window	31.7773	41.8522	41.3997	41.2000	42.0996	37.4609	39.8638	42.9574	36.9009	41.586
Germany	23.2694	34.5788	33.0633	33.0704	36.0738	32.5891	34.5044	37.2342	28.2375	34.9295
Sailboats	31.2055	41.7682	40.7458	40.6497	43.0223	38.8378	40.7782	43.7148	35.9168	41.8173
Sailer	31.1346	41.8023	41.1088	41.0656	41.7161	39.5903	40.3125	42.7598	36.6893	41.8274
Pier	28.6073	39.0873	37.9661	37.8722	40.0244	36.9387	38.5813	40.6892	33.8641	38.3315
Beach	31.6833	43.0056	42.0707	42.1897	43.3411	38.4593	40.4788	44.0251	36.6774	42.8781
Stream	23.73	34.9704	33.5320	33.9208	34.8468	34.2587	33.922	36.2111	30.2308	32.4985
Rafting	28.3803	35.7872	35.2753	34.7022	37.1610	32.6017	34.9244	37.2314	32.4193	36.3718
Face	30.3255	39.3933	38.8815	38.4069	39.5213	35.1821	36.8699	39.7966	33.9731	38.8252
Island	30.1768	43.6206	42.4339	42.0262	43.7698	38.8093	38.7347	44.2015	33.9848	42.5647
Statue	31.7369	41.1726	40.6479	40.4973	41.7746	39.6519	40.04	42.0487	38.2696	40.1238
Art	27.5573	37.1242	36.2833	36.1670	35.5056	34.9067	36.3068	36.893	33.7693	34.8548
Lighthouse1	27.6706	39.7766	38.6658	38.4546	38.4396	36.1558	39.3274	41.3186	33.1143	38.7573
Mustang	29.976	40.4603	39.6348	39.5010	39.8170	37.0458	38.3877	40.7999	35.4269	39.7219
Lighthouse2	27.8809	38.5705	37.3375	37.5056	37.8893	36.1858	38.0689	39.6381	33.2052	37.2376
Barn	29.2773	37.3323	36.8923	36.5303	37.3210	35.4731	37.7111	38.4365	33.2663	37.9025
Parrots	32.9867	42.0001	41.9058	41.1679	39.1794	35.0402	41.2111	42.3329	36.6359	41.6941
Arthouse	26.2003	34.5223	34.5107	33.7442	34.9229	34.0578	34.2513	35.18	31.2927	33.7151
AVERAGE	29.1612	39.2590	38.4404	38.2381	39.2453	36.0122	37.8922	40.4172	34.0577	38.6979

**Table 9:** Local patch PSNR for some very difficult demosaicing problems, i.e. the images shown in figure 17.

	Proposed	POCS [3]	LPA-ICI [5]	Linear [10]	Wavelet [14]	Hybrid [15]	SSDD [8]
Lighthouse 1 (Fence)	41.7 dB	31.8 dB	40.7 dB	31.04 dB	22.7 dB	34.5 dB	34.7 dB
Stream (Bank)	33.5 dB	31.0 dB	33.3 dB	31.23 dB	27.0 dB	28.7 dB	29.3 dB
Artwork (Necklace)	27.3 dB	27.0 dB	27.1 dB	31.10 dB	25.1 dB	24.5 dB	24.5 dB

**Table 10:** Timing (in seconds) for different Matlab implementations of demosaicing algorithms on the 512×768 images of the Kodak data set.

	Bilinear	Proposed with wavelets	Proposed with complex wavelets	DLMMSE [2]
TIME[s]	0.97	0.24 (+3)	0.37 (+3)	21
	POCS [3]/[4]	LPA-ICI [5]	Wavelet [14]	Hybrid [15]
TIME[s]	2.1/0.24	3.4	0.102 (+3)	2.8

## Conclusion

A novel demosaicing method was proposed. The algorithm distinguishes itself by being computationally very efficient, which is made possible through performing demosaicing in the dual-tree complex wavelet packet domain. On the other hand, the demosaicing quality is shown to be on par with existing, but slower demosaicing methods. Through carefully chosen restrictions on the complex wavelet filters, the algorithm performs locally adaptive demosaicing, in a decimated wavelet scheme, using a multi-hypothesis decision scheme to improve performance and avoid artifacts. Finally, we remark that the proposed algorithm can readily, and at almost no extra computational cost, be extended to accomodate state-of-the-art joint wavelet-based denoising + deblurring + demosaicing schemes. This has the potential of allowing for very efficient, but high-quality, processing, perhaps even on mobile devices.
